# Aesthetic Preference for Glossy Materials: An Attempted Replication and Extension

**DOI:** 10.3390/bs11040044

**Published:** 2021-03-26

**Authors:** Paul J. Silvia, Rebekah M. Rodriguez, Katherine N. Cotter, Alexander P. Christensen

**Affiliations:** 1 Department of Psychology, University of North Carolina at Greensboro, P.O. Box 26170, Greensboro, NC 27402-6170, USA; rmrodriguez@uncg.edu; 2 Positive Psychology Center, University of Pennsylvania, Philadelphia, PA 19104, USA; katherinencotter@gmail.com; 3 Penn Center for Neuroaesthetics, University of Pennsylvania, Philadelphia, PA 19104, USA; alexpaulchristensen@gmail.com

**Keywords:** art, aesthetics, gloss, shininess, reflection, attractiveness, evolutionary aesthetics

## Abstract

The psychology of art and aesthetics has a long-standing interest in how low-level features, such as symmetry, curvature, and color, affect people’s aesthetic experience. Recent research in this tradition suggests that people find glossy, shiny objects and materials more attractive than flat, matte ones. The present experiment sought to replicate and extend research on the attractiveness of images printed on glossy and flat paper. To control for several possible confounding factors, glossiness was manipulated between-person and varied with methods that held constant factors like weight, color quality, and resolution. To extend past work, we explored art expertise and Openness to Experience as potential moderators. A sample of 100 adults viewed landscape photographs on either high-gloss photo paper or on identical paper in which a flat, matte spray finish had been applied. Ratings of attractiveness showed weak evidence for replication. People rated the glossy pictures as more attractive than the matte ones, but the effect size was small (*d* = −0.23 [−0.62, 0.16]) and not statistically significant. Attractiveness ratings were significantly moderated, however, by individual differences in the aesthetic appreciation facet of Openness to Experience. When aesthetic appreciation was high, people found the images attractive regardless of condition; when it was low, people strongly preferred the glossy images over the matte ones, thus showing the classic glossiness effect. We conclude with some methodological caveats for future research.

## 1. Introduction

A major strand of thought in the psychology of aesthetics explores how low-level stimulus features—such as symmetry, complexity, prototypicality, color, and curvature—affect people’s emotional experiences [1,2,3,4]. One intriguing low-level feature is *glossiness*: whether objects appear relatively dull or shiny. A handful of studies suggests that people prefer shiny materials, possibly because glossiness connotes water [2]. In an early study, Coss and Moore [5] showed adults a set of different papers that varied in their surface finishes, such as glossy, flat, sandy, and sparkly papers. Semantic differential ratings showed that the glossier papers were experienced as “wetter” and as more appealing. Additionally, in a major series of recent experiments, Meert et al. [6] presented images printed on either glossy or plain paper to children and adults and asked them to rank-order them from most to least attractive and to provide an attractiveness rating. Images printed on glossy paper images were ranked and rated as relatively more attractive.

The effect of glossiness on attractiveness was recently replicated in a study of metal objects—people preferred shiny silver coins over dull ones and mirror-polished copper cylinders over cylinders with brushed or dull surfaces [7]—so the effects are not limited to paper. Nevertheless, paper is interesting in its own right and an important material in everyday aesthetics [8]. The glossiness of paper is used in common consumer products and packaging to grab attention and increase sensory appeal, and consumers appear to have complex associations with the glossiness of product packaging [9,10]. For snack foods, for example, glossy packaging implies greasy contents [11,12], whereas matte packaging implies a product that is less refined and more natural [13].

In the present research, we sought to replicate and extend past research on aesthetic preferences for glossy paper. We made two key procedural changes. First, glossiness was manipulated between-person instead of within-person. All the studies conducted by Meert et al. [6] and Coss and Moore [5] varied glossiness within-person, so their participants viewed both glossy and matte images. This kind of design increases statistical power but also makes glossiness a salient dimension of judgment, especially when people are further asked to rank order the images [6]. It’s unclear if similar effects appear when glossiness is manipulated between-person, which is much less likely to make people explicitly aware of glossiness when making their attractiveness judgments. Second, glossiness was varied while holding constant several potentially confounding factors. Past work has not always been detailed about the physical materials used as stimuli, and glossy and matte papers can vary in many extraneous factors (e.g., weight, brightness, perceived cost) that can affect an image’s attractiveness. Glossy photograph paper, in particular, affords higher image resolution and better color fidelity, so comparing glossy photo paper with basic office paper or card stock will introduce many potential confounds.

Finally, we sought to extend the literature on aesthetic preferences for shiny materials by exploring possible moderators. Many low-level factors that make things more appealing—like symmetry, typicality, color, and curvature—are moderated by individual differences related to the arts. One possible moderator is expertise in the arts, which has widespread effects on how people view, think about, and experience visual art [14,15]. A common finding is that experts are less affected by low-level features, whereas novices are more strongly affected by them [16,17]. Another possible moderator is Openness to Experience, a broad higher-order factor of personality [18]. People high in Openness to Experience value the arts more, spend more time on artistic activities, and have greater engagement in the arts [19,20]. Open people also experience low-level features of art differently than less open people [21]. Art expertise and openness to experience seem like reasonable candidates to examine as moderators, but we did not have specific predictions about moderation, so these analyses were exploratory.

## 2. Materials and Methods

### 2.1. Participants and Design

A total of 113 adults at the University of North Carolina at Greensboro volunteered as part of a research participation option in a psychology course. Several participants were excluded based on non-native English proficiency and on checks of inattentive and careless responding (e.g., having high scores on measures of inattentive responding or giving identical responses to a long string of items [22,23,24]), yielding a final sample of 100 people. This final sample was predominantly young (*M* = 18.9 years, *SD* = 3.31, range from 18 to 48) and female-identifying (73%). People were randomly assigned to one of two between-person conditions—*glossy photos* or *matte photos*—using randomized blocks. Both randomized groups largely reflected the demographics of the final sample (glossy: 73.5% female identifying, age *M* = 18.8 years, *SD* = 1.99, range from 18 to 31; matte: 72.5% female identifying, age *M* = 18.9 years, *SD* = 4.23, range from 18 to 48 years).

### 2.2. Procedure

All participants provided written informed consent. The experiment was conducted in small groups of 1–4 participants, and the surveys were presented and controlled on computers running MediaLab. Random assignment was at the participant level, not the session level, so each session would have a mix of condition assignments. The seating was spaced and partitioned to preclude participants viewing each other’s materials and responses. The study was described as a study of the psychology of visual art, personality, and art experiences. Participants first completed the picture ratings and then completed the measures of personality and art knowledge. The images, surveys, and other research materials are available as Supplementary Material at Open Science Framework: https://osf.io/dbfh5/ (accessed on 25 March 2021).

#### 2.2.1. Glossiness Manipulation

People received a file folder containing four color images of landscapes taken from stock photography databases. The images were professionally printed at 8″ × 10″ size (20.3 × 25.4 cm) on Fuji Film Glossy Quality Dry photographic paper. In the glossy condition, people received the unaltered high-gloss prints. In the matte condition, people received prints in which the glossiness had been diminished via the application of a clear matte spray finish (Rust-Oleum Matte Clear Enamel). The images used in each condition were otherwise identical. This approach—producing all photographs using identical materials and processes and then reducing the glossiness of half of them—ensures that the glossy and matte images were processed and printed identically, thus holding key variables (e.g., color rendering and paper quality) constant.

The experiment software guided participants through viewing and rating the photographs. For the instructions, people read:

“Photography is an important kind of visual art. During this part of the study, we’re interested in people’s aesthetic responses (their impressions and feelings) related to landscape photography. For this part, please open the file folder on your desk. It has four photographs in it. Feel free to pick them up and handle them. For each photograph, you’ll be asked a few questions about your impressions of it.”

The software randomly selected one of the four images, displayed a small thumbnail on the screen, and instructed the participants to “please pull out this photograph, have a look, and think about how you feel about it.” The procedure deliberately showed only a small thumbnail of the image (roughly 20 mm wide) that was large enough to help participants select the physical photograph but not so large so that people could make out much detail. This was intended to ensure that people focused on the physical, printed photographs, not the digital image, when making their judgments.

People were free to handle and inspect the photograph for as long as they wished, in part because handling objects increases the accuracy of glossiness perception [25]. After handling and viewing each photograph, they rated it on several dimensions:

- How ATTRACTIVE is this photograph? (1 = *Not at all attractive*, 7 = *Very attractive*).
- How APPEALING is this photograph? (1 = *Not at all appealing*, 7 = *Very appealing*).
- How INTERESTING is this photograph? (1 = *Not at all interesting*, 7 = *Very interesting*).
- How COLORFUL is this photograph? (1 = *Not at all colorful*, 7 = *Very colorful*).
- What is your impression of the OVERALL QUALITY of this photograph? (1 = *Very low quality*, 7 = *Very high quality*).

These items stem from our prior work on the aesthetics of metal objects [7] as well as prior studies of glossy paper. The key outcome in past work was ratings of attractiveness [6], so it was our primary outcome measure. The other items were included to explore the potential breadth of glossiness effects. People completed these items in this order for all four images.

#### 2.2.2. Measures of Individual Differences

Art knowledge and expertise were measured with two scales. The *aesthetic fluency scale* [26] is a popular scale for measuring people’s knowledge about the arts [27,28,29]. It presents 10 figures and ideas from art history (e.g., Fauvism, Isamu Noguchi, Mary Cassatt) and asks people to report their knowledge of it on a 5-point scale ranging from 0 (*I have never heard of this artist or term*) to 4 (*I can talk intelligently about this artist or idea in art*). The scores are summed for an overall score (Cronbach’s α = 0.84). The *art experience questionnaire* [30] asks 8 questions about people’s formal training in the arts (e.g., classes taken in art history) and how often they engage in activities related to the visual arts (e.g., visiting museums and reading about art) on 6-point and 7-point scales. The scores were summed for an overall score (α = 0.73).

Personality traits were measured with the HEXACO-100 [31], which assesses 6 higher-order traits: Honesty-Humility, Emotionality, eXtraversion, Conscientiousness, Agreeableness, and Openness to Experience [32]. Our primary interest was in Openness to Experience, given its deep links to artistic knowledge, interest, and engagement [19]. The items are completed on a 5-point scale (1 = *strongly disagree*, 5 = *strongly agree*). The HEXACO-100 provides an overall Openness to Experience score (16 items; α = 0.83) as well as four facet scores (4 items each): Aesthetic Appreciation (α = 0.65), Creativity (α = 0.73), Inquisitiveness (α = 0.62), and Unconventionality (α = 0.56). The Aesthetic Appreciation facet was the key facet for our purposes. In addition, we included the Openness to Experience subscale of the Big Five Aspects Scale (BFAS [33]), which includes 20 items, completed on a 5-point scale (1 = *strongly disagree*, 5 = *strongly agree*), measuring the aspects of Openness (α = 0.74) and Intellect (α = 0.79). These two aspects are broader and more heterogeneous than the HEXACO facets [18,34]. The Openness aspect captures engagement in imaginative, aesthetic, emotional, and creative experiences; the Intellect aspect captures engagement in cognitive and intellectual experiences.

## 3. Results

### 3.1. Data Preparation and Scoring

The data were screened and coded using R 4.0 [35] and analyzed in Mplus 8.1. We conducted regression models estimated with maximum likelihood and robust standard errors. Descriptive statistics for all outcomes are shown in Table 1.

### 3.2. Main Effects of Glossiness

Did participants rate the attractiveness of images in the glossy group differently than they rated images in the matte group? As the predictor is categorical (glossy = 0, matte = 1) and the outcomes are Likert-scale ratings, we report *Y*-standardized regression coefficients. These represent the difference, in the outcome’s *SD* units, between the two groups [36], so the regression coefficients represent effect sizes in the Cohen’s *d* metric (small = 0.20, medium = 0.50, large = 0.80 [37]).

**Figure 1:**
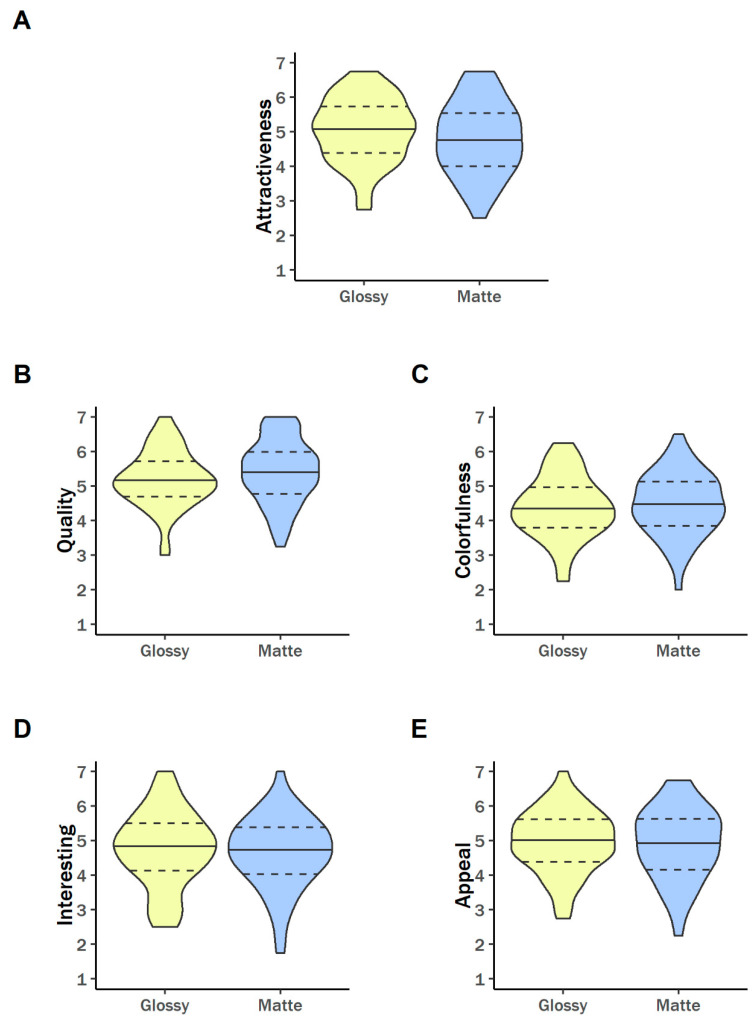
Effects of glossy versus matte paper on the outcome variables, which were ratings for the images’ (**A**) attractiveness, (**B**) quality, (**C**) colorfulness, (**D**) interestingness, and (**E**) appeal.

As depicted in Figure 1A, photos with a glossy finish received higher attractiveness ratings than photos with a matte finish—the same direction as past work—but the difference was not significant, *b* = −0.23 [−0.62, 0.16], *SE* = 0.20, *p* = 0.250, with an effect size in the “small” range. Glossiness had the opposite effect on perceived image quality (see Figure 1B). Although participant ratings of quality did not differ significantly between glossy and matte photos (*b*= 0.25 [−0.14, 0.63], *SE* = 0.20, *p* = 0.203), raters judged photographs with a matte finish as being of slightly higher quality than photographs with a glossy finish, again with a small effect size. For the remaining ratings—colorfulness (*b* = 0.09 [−0.30, 0.48], *SE* = 0.20, *p* = 0.644; see Figure 1C), interest (*b* = −0.09 [−0.48, 0.30], *SE* = 0.20, *p* = 0.658; see Figure 1D), and appeal (*b* = −0.06 [−0.45, 0.33], *SE* = 0.20, *p* = 0.746; see Figure 1E)—the differences between the glossy and matte conditions were very small and non-significant.

In sum, little evidence was found for replication of the core finding from past work. The main effect of glossiness was in the same direction but small in effect size and not statistically significant.

### 3.3. Exploring Moderators of Attractiveness

Our next step was to explore possible moderators of the main effect of glossiness on attractiveness ratings, again using regression models in Mplus. The moderators, shown in Table 2, were the measures of art expertise (the aesthetic fluency scale and the art experience questionnaire) and Openness to Experience and their facets. The predictors were centered at the sample’s grand mean, and an interaction term was created from the centered variables [38].

**Figure 2:**
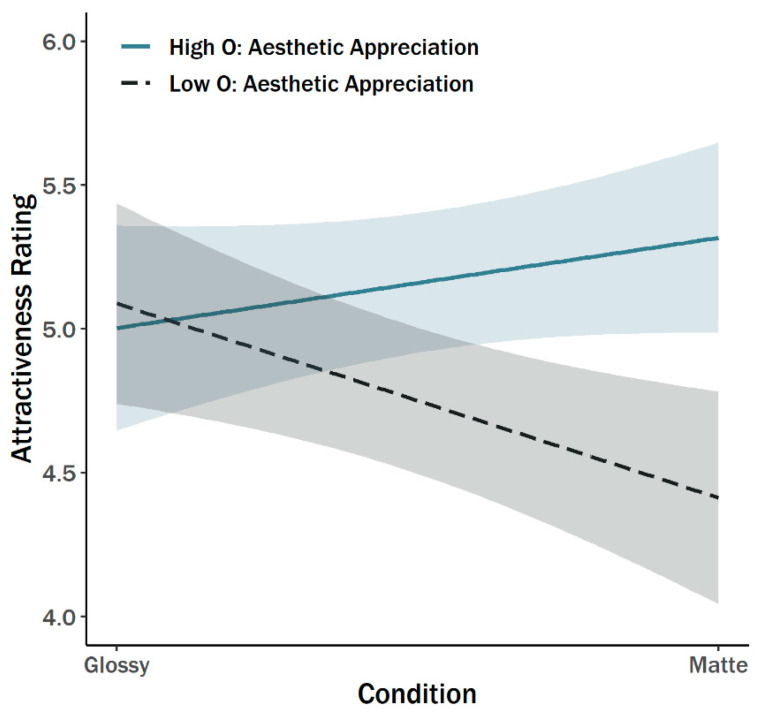
Interaction of paper glossiness and the HEXACO Aesthetic Appreciation facet on attractiveness ratings. (Note. The bands around the regression lines are 95% confidence intervals.).

As illustrated in Table 2, nine separate interaction models were examined for their potential moderating effects on attractiveness ratings. Of these models, the Aesthetic Appreciation component of the Openness to Experience HEXACO factor had the strongest interaction effect, *b* = 0.56 (*SE* = 0.19), *p* = 0.003, model *R*^2^ = 0.12, as well as the only statistically significant interaction. Figure 2 displays the pattern. People high in aesthetic appreciation found the photos attractive regardless of glossiness, but people low in aesthetic appreciation showed a pronounced preference for glossy over matte photos.

## 4. Discussion

In the present research, we sought to replicate and extend past research on the effects of glossy paper materials on aesthetic preferences [5,6]. Regarding replication, at best weak evidence for replication was found. For ratings of the attractiveness of glossy vs. matte paper, the effect was in the right direction, but the effect size was small and not statistically significant (*d* = −0.23 [−0.62, 0.16]).

We think a few factors are likely behind the diverging results. The first is sampling variability, of course, which plays a large but difficult to discern role in all research [39]. Beyond that, our smaller effect likely results from using a between-person manipulation. Within-person and between-person manipulations have well-known strengths and trade-offs. Aside from their higher power, within-person manipulations that expose participants to all levels of a glossiness variable could heighten the salience of glossiness as a parameter of the stimuli, especially when the participants are asked to rank-order the stimuli [6]. The between-person manipulation, however, cannot adjust for mean-level differences, such as the tendency for some people to give higher attractiveness ratings overall. It’s also possible that our manipulation of glossiness was relatively mild. To control for several extraneous factors, we created matte images by modifying glossy ones. This method keeps paper weight, color quality, and similar factors constant, but it may be a relatively subtle manipulation. It would be worthwhile for future research to evaluate the role of possible contrast effects by using both between-person and within-person manipulations within the same sample of participants. Finally, it is difficult to control for likely between-study differences in environmental illumination, which could influence the relative distinctiveness of flat and shiny finishes. Manipulating illumination within a single study would be a fruitful task for follow-up research. 

Our second goal was to extend the literature by exploring individual-differences moderators. Evidence for moderation was limited to aesthetic appreciation, a facet of Openness to Experience associated with valuing and engaging with the arts. People high in the aesthetic appreciation facet are more interested in the arts and more attuned to beauty in both the arts and in the natural world [31], so it is not surprising that the facet most closely linked to aesthetic perception and experience emerged as a significant moderator. Thus, the moderation effect of Openness to Experience was driven by the aesthetic appreciation characteristic and not the trait as a whole, which supports contemporary trends in personality research that go beyond trait-level associations and focus on lower level facets [40,41].

People high in aesthetic appreciation were insensitive to the glossiness manipulation—they gave high attractiveness ratings in both conditions. However, people low in aesthetic appreciation showed the classic glossiness effect—they rated the glossy images as more attractive than the matte images. This pattern is consistent with moderation effects in other areas of aesthetics research. A common finding is that art novices are more strongly affected by low-level stimulus features—such as when novices’ liking is more strongly affected by symmetry—whereas art experts are less influenced by surface features and more sensitive to formal, conceptual, and historical features [14,16,17,42].

The evidence for moderation is consistent with past work, but the fact that only one facet of Openness to Experience—albeit one closely linked to the arts—showed significant moderation suggests that the interaction results should be viewed as tentative pending future research. Likewise, research on art expertise does not always find diminished sensitivity to low-level features. In the study of curvature, people and related species prefer rounded, curved objects over angular, jagged ones [3,43,44], but most studies on experts have found that experts have an even greater preference for curved objects [45,46,47]. Thus, although the results of the current study were limited, they support the notion that the roles of expertise and personality in preferences for glossy materials deserve more attention in future research.

## Figures and Tables

**Table 1:** Descriptive statistics.

Outcome	Glossy Images	Matte Images	Effect Size (*d*)
Attractive	5.04 (0.94)	4.81 (1.05)	−0.23 [−0.62, 0.16]
Appealing	4.97 (0.91)	4.91 (1.04)	−0.06 [−0.45, 0.33]
Interesting	4.39 (0.90)	4.47 (.91)	−0.09 [−0.48, 0.30]
Colorful	4.76 (1.10)	4.66 (1.04)	0.09 [−0.30, 0.48]
Quality	5.20 (0.81)	5.42 (0.93)	0.25 [−0.14, 0.63]

Note. The sample size is *n* = 49 (glossy) and *n* = 51 (matte). Standard deviations are in parentheses. The effect size *d* is estimated from a regression model using maximum likelihood with robust standard errors. Negative signs reflect larger values for the glossy condition.

**Table 2:** Interactive effects of glossiness and individual differences on attractiveness.

Moderator	Glossy Main Effect	Moderator Variable’s Main Effect	Interaction	Model *R*^2^
O: Aesthetic Appreciation	−0.18 (0.19), *p* = 0.328	**0.24 (0.09)**, ***p* = 0.012**	**0.56 (0.19)**, ***p* = 0.003**	0.12
O: Inquisitiveness	−0.13 (0.20), *p* = 0.501	0.21 (0.14), *p* = 0.138	−0.08 (0.28), *p* = 0.788	0.04
O: Creativity	−0.22 (0.19), *p* = 0.264	0.06 (0.12), *p* = 0.613	0.11 (0.23), *p* = 0.627	0.02
O: Unconventionality	−0.19 (0.19), *p* = 0.337	0.17 (0.16), *p* = 0.299	−0.05 (0.33), *p* = 0.890	0.03
HEXACO O	−0.14 (0.19), *p* = 0.448	**0.32 (0.16)**, ***p* = 0.049**	0.37 (0.32), *p* = 0.238	0.06
BFAS Openness	−0.18 (0.20), *p* = 0.336	**0.39 (0.17)**, ***p* = 0.020**	−0.12 (0.34), *p* = 0.733	0.06
BFAS Intellect	−0.23 (0.20), *p* = 0.236	0.28 (0.22), *p* = 0.196	−0.14 (0.43), *p* = 0.744	0.04
Aesthetic Fluency	−0.22 (0.20), *p* = 0.270	0.01 (0.02), *p* = 0.572	0.02 (0.04), *p* = 0.677	0.02
Art Experience	−0.15 (0.19), *p* = 0.432	**0.04 (0.02)**, ***p* = 0.014**	0.04 (0.03), *p* = 0.273	0.06

Note. Statistically significant effects are in bold. The regression coefficients are unstandardized. Glossiness is coded 0 (glossy) and 1 (matte). Moderators preceded by “O”: are individual components of the HEXACO O facet. The only significant interaction—aesthetic appreciation—is depicted in Figure 2.

## Data Availability

The data and associated files are publicly available at Open Science Framework: https://osf.io/dbfh5/ (accessed on 25 March 2021).

## Supplementary Materials

The images, surveys, and other research materials are available at Open Science Framework: https://osf.io/dbfh5/ (accessed on 25 March 2021).
